# Clinical outcome of alveolar ridge augmentation with individualized CAD-CAM-produced titanium mesh

**DOI:** 10.1186/s40729-017-0097-z

**Published:** 2017-07-26

**Authors:** K. Sagheb, E. Schiegnitz, M. Moergel, C. Walter, B. Al-Nawas, W. Wagner

**Affiliations:** 1 Department of Oral and Maxillofacial Surgery, Plastic Surgery, University Medical Centre of the Johannes Gutenberg-University Mainz, Mainz, Germany; 2 Mediplus, Oral and Maxillofacial Surgery, Private Praxis, Mainz, Germany

**Keywords:** CAD-CAM, Titanium mesh, Augmentation, Bone atrophy, Bone regeneration, PRF

## Abstract

**Background:**

The augmentation of the jaw has been and continues to be a sophisticated therapy in implantology. Modern CAD-CAM technologies lead to revival of old and established augmentation techniques such as the use of titanium mesh (TM) for bone augmentation. The aim of this retrospective study was to evaluate the clinical outcome of an individualized CAD-CAM-produced TM based on the CT/DVT-DICOM data of the patients for the first time.

**Methods:**

In 17 patients, 21 different regions were augmented with an individualized CAD-CAM-produced TM (Yxoss CBR®, Filderstadt, Germany). For the augmentation, a mixture of autologous bone and deproteinized bovine bone mineral (DBBM) or autologous bone alone was used. Reentry with explantation of the TM and simultaneous implantation of 44 implants were performed after 6 months. Preoperative and 6-month postoperative cone beam computed tomographies (CBCT) were performed to measure the gained bone height.

**Results:**

The success rate for the bone grafting procedure was 100%. Thirty-three percent of cases presented an exposure of the TM during the healing period. However, premature removal of these exposed meshes was not necessary. Exposure rate in augmentations performed with mid-crestal incisions was higher than in augmentations performed with a modified poncho incision (45.5 vs. 20%, *p* = 0.221). In addition, exposure rates in the maxilla were significantly higher than in the mandible (66.7 vs. 8.3%, *p* = 0.009). Gender, smoking, periodontal disease, gingiva type, used augmentation material, and used membrane had no significant influence on the exposure rate (*p* > 0.05). The mean vertical augmentation was 6.5 ± 1.7 mm, and the mean horizontal augmentation was 5.5 ± 1.9 mm. Implant survival rate after a mean follow-up of 12 ± 6 months after reentry was 100%.

**Conclusion:**

Within the limits of the retrospective character of this study, this study shows for the first time that individualized CAD-CAM TM provide a sufficient and safe augmentation technique, especially for vertical and combined defects. However, the soft tissue handling for sufficient mesh covering remains one of the most critical steps using this technique.

## Background

Dental implant placement is an effective treatment method for the replacement of lost teeth with high survival rates after long-term follow-up [1–3]. However, the long-term success and stability of implants in function are directly correlated with the quality and quantity of the available bone at the prospective implant site [4, 5]. Despite the development of various techniques and augmentation materials, the reestablishment of an adequate amount of bone especially in the vertical direction remains challenging. Many different augmentation procedures, depending on location and size of the defect, were described and have been studied extensively in human and animal studies by evaluating healing events via histological, radiological, and clinical outcomes [6].

The use of conventional titanium meshes (TM) was first described for the reconstruction of osseous-maxillo-facial defects and secondarily introduced for osseous restoration of deficient edentulous maxillary ridges [7–9]. In addition, they were used for localized alveolar ridge augmentation of ridge defects with simultaneous and secondary implant insertion [10–12]. Further clinical studies showed predictable results for both lateral and vertical bone reconstruction with this titanium mesh technique [13]. These conventional TM are designed as planar plates. Therefore, intraoperative manual shaping and bending of the premade TM according to the individual defect is necessary, which is manually challenging and time-consuming [14, 15]. Furthermore, the corners and edges of these cut and bended meshes possibly provoke damages to the gingiva and mesh exposure. The CAD-CAM technology provides a sufficient solution for these disadvantages. Based on the cone beam computed tomography (CBCT) scan data of the bony defect and a digital work flow system, individualized titanium mesh cages can be created that it can fit perfectly over the bone defect of the augmentation site. However, due to the stiffness of the TM with mechanical irritation to the mucosal flap, the risk of flap dehiscence with exposure of the graft and possible particular or even complete loss of the graft material remains [16, 17].

The aim of this clinical study was to present the clinical outcome of individualized CAD-CAM-produced TM in combination with particulate autogenous bone mixed with deproteinized bovine bone mineral (DBBM) used to augment horizontal and/or vertical bony defects in both maxillary and mandibular arches, within a two-stage technique. Furthermore, gained horizontal and vertical bone height and the influence of incision technique, location, and reason of bone defect on dehiscence rate and augmentation success were evaluated.

## Methods

### Study design

In a retrospective study, the clinical outcome of an individualized CAD-CAM-produced TM (Yxoss CBR®, Filderstadt, Germany) inserted by experienced surgeons in the Department of Oral and Maxillofacial Surgery of the University Medical Centre Mainz, Germany, between December 2014 and January 2017, was analyzed. Therefore, all patients with this CAD-CAM mesh augmentation and reentry operation for implant insertion in this time period were included in this study. There were no patients excluded from this study. The retrospective data analysis was conducted in accordance with the Helsinki Declaration of 1975, as revised in 2008, and all patients signed an informed consent. After consulting the local ethic committee, the decision was that due to the retrospective character of this study with no additional data acquisition, no ethical approval was needed according to the hospital laws of the appropriate state (Landeskrankenhausgesetz Rhineland Palatinate, Germany).

### Surgical procedure

**Fig. 1:**
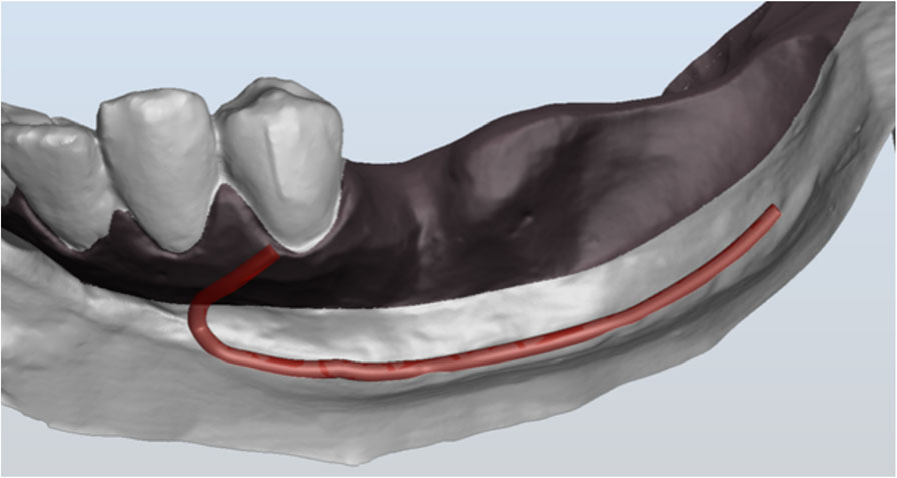
Schematic drawing of the poncho flap approach

**Fig. 2:**
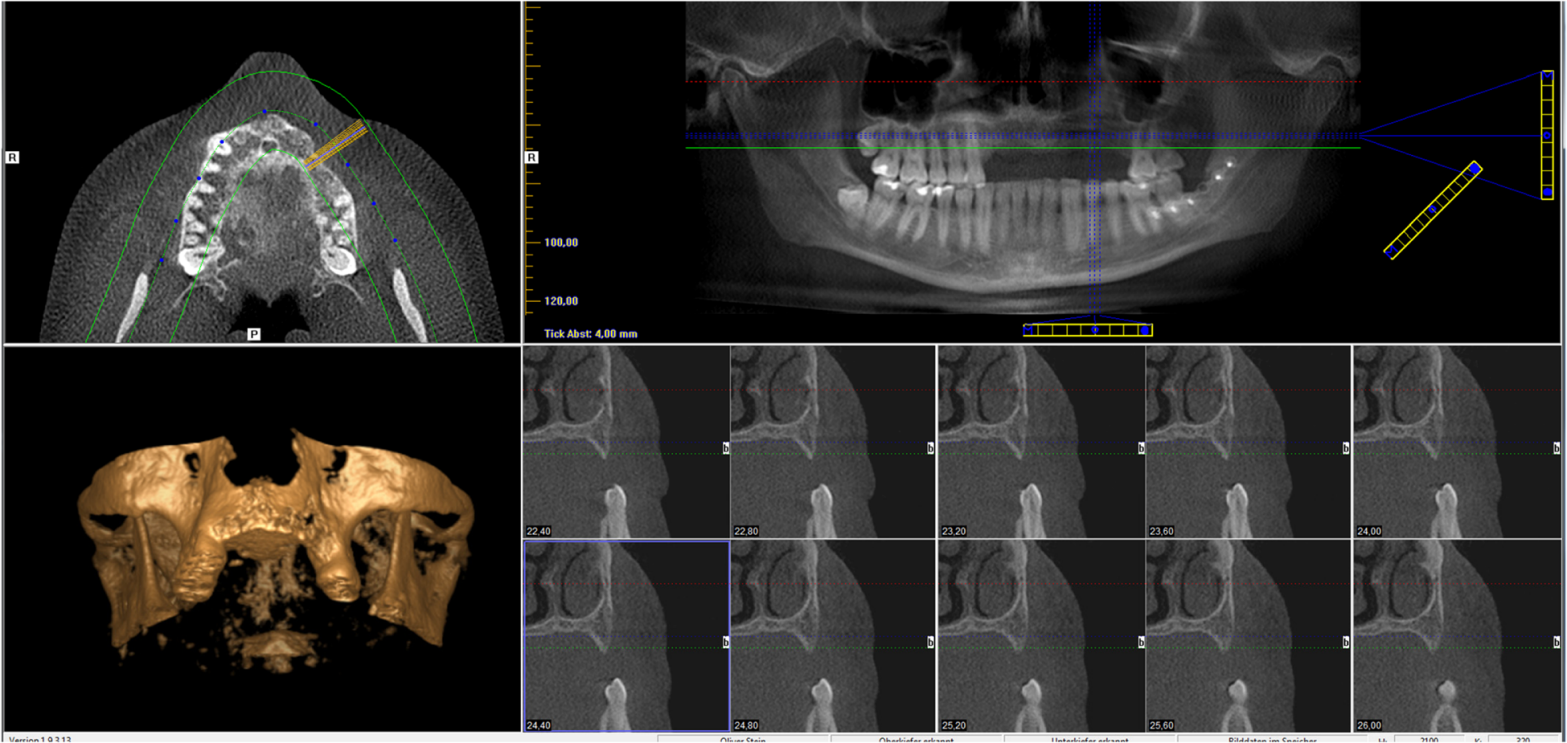
Preoperative CBCT scan showing the vertical and horizontal deficit

**Fig. 3:**
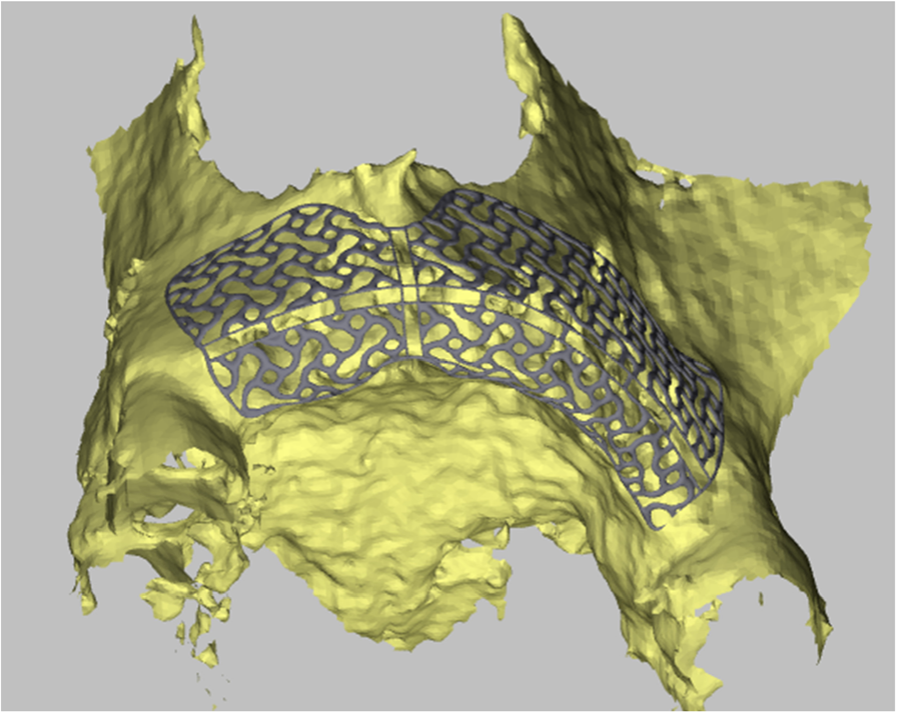
3D design of the CAD-CAM-based individualized TM by ReOss®

**Fig. 4:**
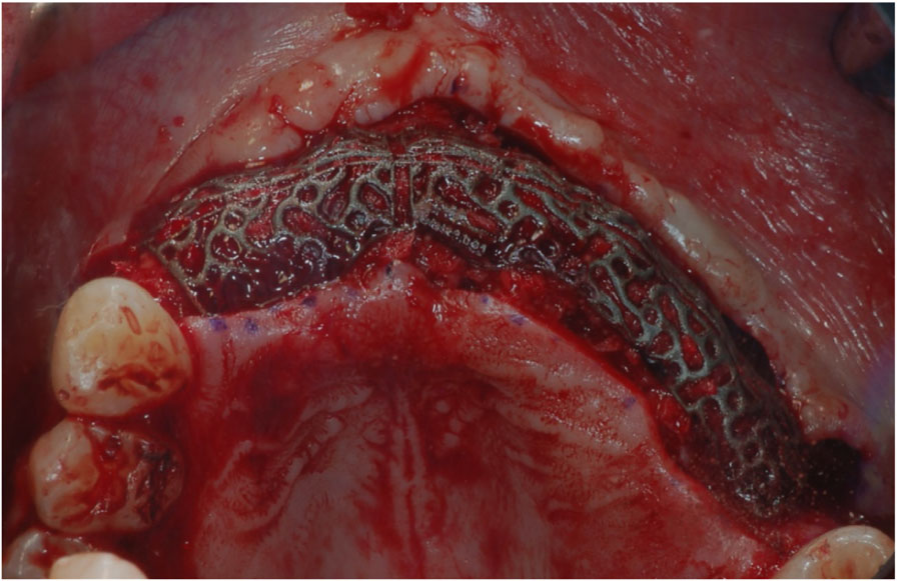
Intraoperative clinical picture after insertion of CAD-CAM mesh

**Fig. 5:**
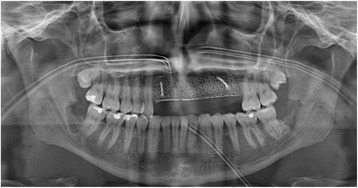
Orthopantomogram after augmentation

**Fig. 6:**
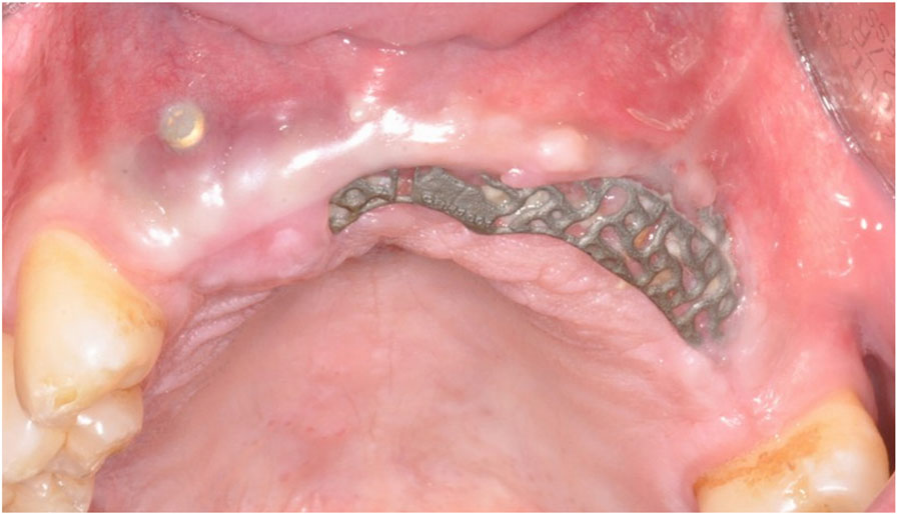
Clinical picture after 6 months showing an exposure of the mesh

**Fig. 7:**
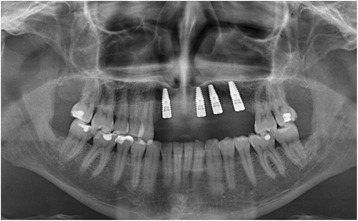
Orthopantomogram after implant insertion

**Fig. 8:**
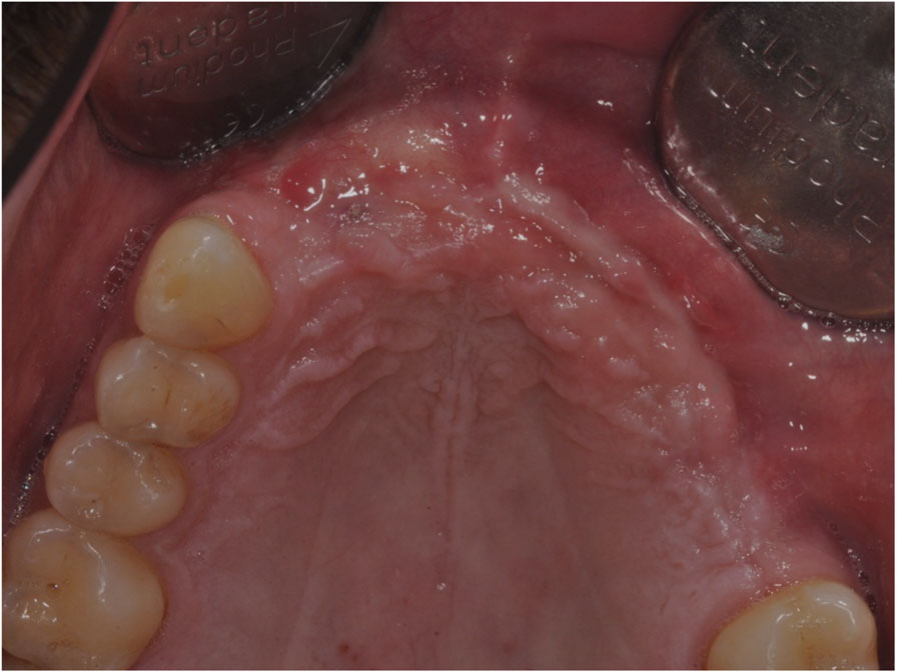
Clinical picture after implant insertion

With the Customized Bone Regeneration (CBR®) technology, the manufacturing of custom-molded protective TM is achieved. Using the DICOM data of the CBCT scan of the defect region, an individualized mesh was produced using the CAD-CAM technology by ReOss Ltd. (Filderstadt, Germany). The meshes were produced using three-dimensional printing. Surgeries were performed under local or anesthesia or in general anesthesia. Depending on the defect configuration, a mid-crestal or a modified poncho incision was performed. For the modified poncho, the incision was made in the vestibulum parallel to the alveolar ridge by a tunneling preparation (Fig. 1). This poncho technique was preferred in pronounced vertical defects. After incision, preparation of a mucoperiosteal flap, debridement of scar tissue, and exposure of the defect were conducted. Then, a passive tension-free fit of the TM was verified. Autologous bone was harvested with bone scraper from the intraoral regions, such as the tuber maxillae, the symphysis, the mandibular body, and the retromolar pad region (Safescraper®, Zimmer Biomet, Germany) or from the iliac crest. The TM was loaded with an equal mixture of deproteinized bovine bone mineral (Bio-Oss®, Geistlich Biomaterials, Switzerland) and autologous bone or autologous bone alone and fitted to the defect. The rationale of mixing autogenous bone with DBBM is to combine the scaffold properties of the xenograft with the osteogenic and osteoinductive properties of the autograft [18–20]. To fix the TM in place, two bone screws were used. TM were covered in situ with nothing, a resorbable collagen membrane (Bio-Gide®, Geistlich Biomaterials, Switzerland) alone, or a resorbable collagen membrane, followed by platelet-rich fibrin (PRF) membranes (Choukroun A-PRF™) in a double-layer technique. The PRF membranes were produced according to the manufacturer’s protocol. All patients underwent an oral antibiotic therapy with amoxicillin 1000 mg (1 to 0–1) for 5 days starting at the operation day. Reentry with explantation of the TM and simultaneous implantation were performed after a 6-month healing period. Figures 2, 3, 4, 5, 6, 7, and 8 present a clinical case.

### Radiographic analysis

**Fig. 9:**
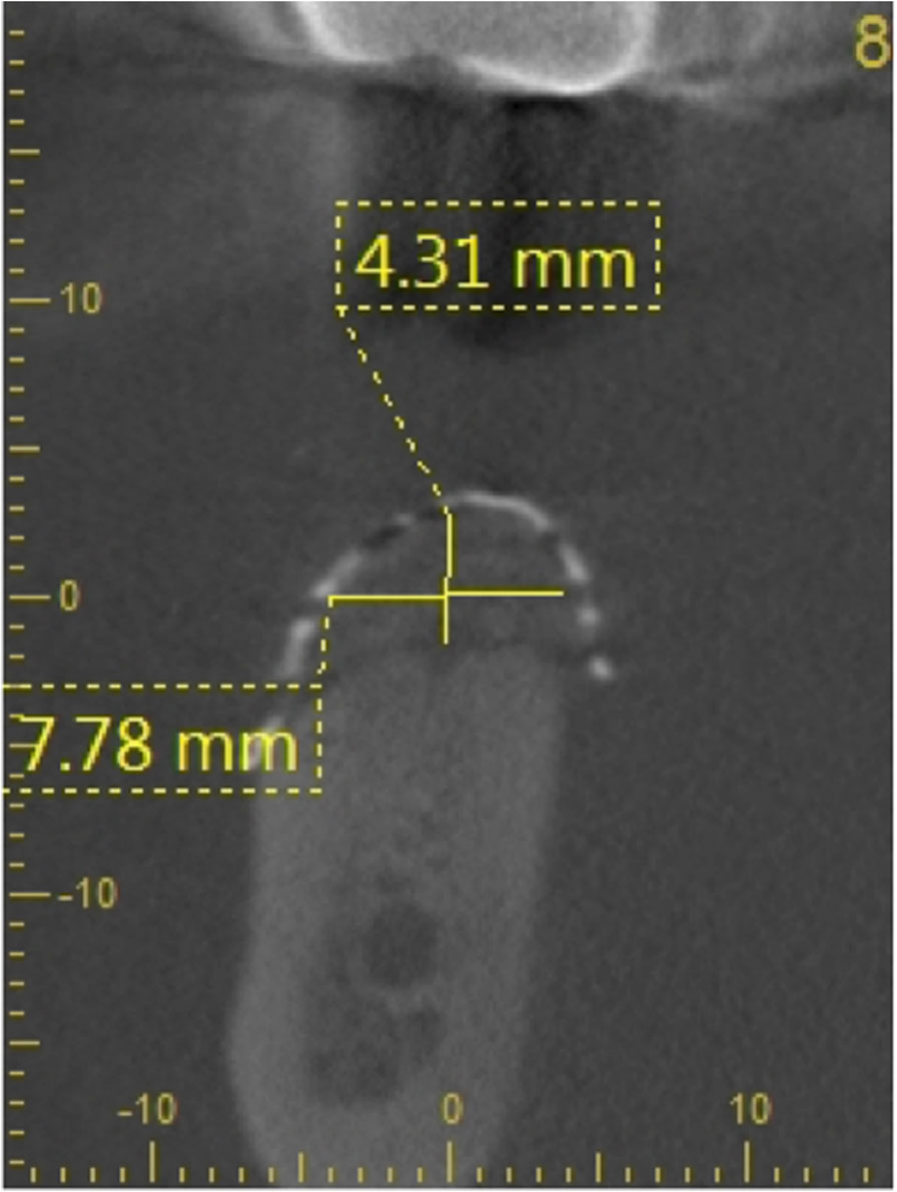
Representative picture of analysis of vertical and horizontal bone augmentation on CBCT scan

Cone beam computed tomography (CBCT) of the treated sites was performed before augmentation procedure and 6 months postoperatively at time of reentry. Craniofacial bone and TM showed different radio-opacity, which allowed their easy differentiation on the scans after regulating the brightness and contrast. In our department, two different CBCTs were available (Accuitomo, J. Morita Corporation, Japan and 3D eXamVision, Kavo Dental GmbH, Germany). For large defects and in existence of possible other indications (e.g., sinus maxillary diagnostic), the 3D eXamVision was used. Small locoregional defects were imaged with the Accuitomo. Gained bone height and width was quantified using the KaVo-eXam Vision software (Kavo Dental GmbH, Germany) and One Volume Viewer software (J. Morita Corporation, Japan) on one descriptive slide of the CBCT scan [21]. Therefore, the margins of the basal and grafted bone and the rim of the TM were defined, and linear measurements for vertical and horizontal bone augmentation were made on one descriptive coronal section in a midalveolar position (Fig. 9). For horizontal bone augmentation, the widest horizontal distance in midalveolar position was evaluated. However, this evaluation technique has to be assessed critically as it is hard to distinguish between graft material and real new bone. A layer of soft tissue with some embedded granules underneath the mesh, which is usually removed at the time of implant insertion and mesh removal, could not be subtracted from the augmentation bone gain regularly.

### Statistics

The statistical analysis was performed using the IBM® SPSS® Statistics version 23.0 for Windows®. We report descriptive *p* values of tests, and no adjustment to multiple testing due to the low case number was performed. Chi-quadrat-test was performed to identify potential influencing factors for a higher risk of exposure of the TM.

## Results

### Patient data

In the investigated time period, 17 patients received 21 TM augmentations. Fourteen of these patients were women and three men. Mean age at the time of augmentation was 37 ± 15 years (17–64 years). Twelve of the patients were non-smoker, and 5 patients were smoker. In 8 patients, a steady periodontal disease could be detected. Sixty-five percent (*n* = 11) of the patients presented a thin gingival morphotype A and 35% (*n* = 6) of the patients a thick gingival morphotype B. Fifty-seven percent (*n* = 12) of the augmented regions were located in the mandible and 43% (*n* = 9) in the maxilla. The length of the defects ranged from a minimum of one to a maximum of nine teeth (mean 3 ± 2 teeth). A mid-crestal incision was performed in 11 augmentation sides (52%), and a modified poncho incision was applied in 10 augmentation sides (48%). In 19 augmented sites, a mixture of deproteinized bovine bone mineral and autologous bone was used. In two augmented sites, autologous bone alone was inserted. In two cases, no membrane for covering the TM was used, in six cases, a resorbable collagen membrane, and in 13 cases, a double layer of collagen membrane, and PRF membranes were inserted.

### Clinical and radiological outcome

**Fig. 10:**
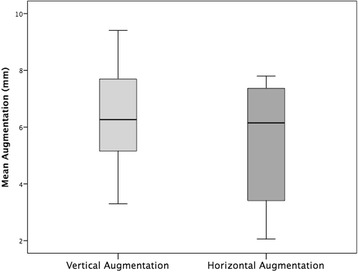
Mean vertical and horizontal augmentation height (mm)

In all cases, the individualized TM could easily be placed into the planned area of augmentation. The postoperative healing was uneventful in 14 cases (67%) during the follow-up time of 6 months until reentry. In seven cases (33%), an exposure of the TM after a period ranging from 5 to 12 weeks from first-stage surgery was seen. All the dehiscences appeared in the area of the suture. Patients with TM exposure were treated with chlorhexidine mouthwash rinse. The premature removal of the TM after exposure was necessary in none of the cases, and all the preoperatively planed implantations could be carried out. Therefore, exposure of the TM had no negative influence on the clinical outcome of the augmentation procedure and success of the bone grafting procedure was 100%. Exposure rate in augmentations performed with mid-crestal incisions (45.5%) was higher than in augmentations performed with a modified poncho incision (20%), however, not statistically significant (*p* = 0.221). In addition, exposure rates in the maxilla were significantly higher than in the mandible (66.7 vs. 8.3%, *p* = 0.009). Gender, smoking, periodontal disease, gingiva type, used augmentation material, and used membrane had no significant influence on exposure rates (*p* > 0.05). Comparing the preoperative and 6-month postoperative cone beam computed tomography (CBCT), a mean vertical augmentation of 6.5 ± 1.7 mm and a mean horizontal augmentation of 5.5 ± 1.9 mm was achieved (Fig. 10). After a mean follow-up of 12 ± 6 months after second-stage surgery, none of the 44 inserted implants was lost, indicating a survival rate of 100%.

## Discussion

The vertical and horizontal regeneration of resorbed alveolar ridges remains a challenging surgical procedure, especially in the case of extensive bone atrophy. During the past years, different augmentation techniques have been proposed to restore an adequate bone volume. The aim of this study was to evaluate a technique for ridge augmentation in the maxilla and mandible using an individualized CAD-CAM-produced titanium mesh.

In our study, titanium mesh exposure occurred in 33% of the augmented sites. However, a premature removal of these exposed meshes was not necessary in any of these cases. In addition, exposure of the mesh did not affect the final outcome of the augmentation as implant insertion was possible in all cases in the desired position, indicating a success rate of 100% for the augmentation technique. The titanium mesh exposure is a common complication, with reported exposure rates between 0 and 80% in the international literature [11, 17]. Sumida et al. investigated custom-made titanium devices for bone augmentation compared to conventional titanium meshes in 26 patients [15]. Mucosal rupture occurred in a patient in the custom-made group (7.7%) and 3 in the conventional group (23.1%), indicating better results for the individualized mesh, however, neither statistically nor clinically significant. In a further clinical study investigating a conventional titanium mesh, exposure occurred in 6 of the 17 patients (35%) [22]. Two of them were early exposures (within 2 weeks) and 4 of them late exposures. Corinaldesi et al. showed an exposure rate of 14.8% [23]. These cases necessitated premature removal of the titanium mesh. In these exposed sites, reduction in mean bone regeneration was observed compared to mesh-retained sites in patients who received simultaneous augmentation and implant placement. A critical point discussed in the international literature is the time elapsed between augmentation procedure and exposure. An early exposure within the first weeks showed a negative impact on bone regeneration in contrast to a late exposure [12, 23–25]. To prevent such an exposure, an accurate soft tissue handling in terms of tension-free flaps over the mesh is mandatory.

In our study, PRF membranes were additionally to collagen membranes used to cover the CAD-CAM mesh. The aim of this clinical approach was to improve and accelerate wound healing. The results with the low exposure rates and the sufficient augmentation heights indicated that these PRF membranes are a promising technique. However, due to low case number in the control group without a PRF membrane, definitive conclusions are not possible. The positive effects of PRF regarding wound healing may be explained by the contents of the PRF clot. These clots contain stem cells, fibrin, platelets, and leucocytes [26, 27]. Furthermore, PRF membranes have a sustained release of high quantities of the growth factors TGFbeta-1, PDGF-AB, and VEGF and coagulation matricellular glycoprotein (thrombospondin-1, TSP-1) during 7 days [27]. Therefore, PRF is a biodegradable scaffold that promotes the development of microvascularization and epithelial cell migration to its surface [28, 29]. There are several clinical studies and systematic reviews that show the promising potential of PRF for bone and soft tissue regeneration [28, 30, 31]. Torres et al. examined the effect of platelet-rich plasma in preventing mesh exposure by using it to cover conventional meshes [32]. In this study, 43 alveolar bone augmentations with the mesh technique using anorganic bovine bone as graft material were performed. In half of the patients, the meshes were covered with platelet-rich plasma, whereas in the other half, the meshes were not. The results showed that mesh exposure was significantly less in the platelet-rich plasma group as well as that bone augmentation was higher in the platelet-rich plasma group than in the control group. In conclusion, these results promote the use of PRP/PRF in augmentation procedures.

Besides the use of membranes, the application of a sufficient incision technique is crucial to avoid dehiscences. In our study, augmentations performed with a modified poncho incision had lower exposure rates than augmentations performed with a mid-crestal incision. All the dehiscences appeared in the area of the suture. Therefore, positioning the margin of the wound in the vestibulum and in distance to the mesh seems to reduce the risk for an exposure of the TM as the margin of a wound represents the most important nutritional structure for survival and the basis for reliable wound healing [33, 34]. In addition, exposure rate in the maxilla was significantly higher than in the mandible. This could be explained with the higher augmentations in the maxilla in our study. In our study, both craniofacial and iliac crest bones were used for augmentation procedures. This may influence later bone resorption and long-term stability. However, a recent influence of the used material on augmentation success was not seen.

The results showed that in all 21 augmented sites, a significant ridge augmentation was achieved, with a mean vertical augmentation of 6.5 ± 1.7 mm and a mean horizontal augmentation of 5.5 ± 1.9 mm. To our best knowledge, this is the first study investigating these parameters in individualized CAD-CAM-produced titanium meshes. For conventional titanium meshes, several studies were published. Torres et al. investigated the effectiveness of anorganic bovine bone in alveolar bone augmentation with the titanium mesh technique [32]. The average bone height gained was 3.3 ± 0.2 mm and the average bone width 3.9 ± 0.2 mm. Corinaldesi et al. indicated in 24 patients with 27 micromeshes a mean vertical bone augmentation of 5.4 ± 1.81 mm [23]. Pieri et al. examined the clinical and radiographic parameters of implants placed in augmented ridges using a 70:30 mixture of autogenous bone and bovine bone mineral in association with titanium meshes [21]. Radiographic assessment showed a mean vertical augmentation of 3.71 ± 1.24 mm and mean horizontal augmentation of 4.16 ± 0.59 mm. Proussaefs and Lozada applied a titanium mesh for localized alveolar ridge augmentation with an equal mixture of autogenous bone and bovine bone mineral [22]. Radiographic evaluation indicated a mean vertical ridge augmentation of 2.56 ± 1.32 mm and a mean horizontal ridge augmentation of 3.75 ± 1.33. In total, the mesh technique is a predictable procedure with sufficient horizontal and vertical bone gain.

## Conclusions

Within the limitations of this study, being retrospective and having no control group, the results show that individualized CAD-CAM-produced titanium meshes are a safe and predictable procedure for large vertical and horizontal ridge augmentations. The soft tissue covering remains one of the most critical steps using this technique. However, exposure of the mesh does not result in complete loss of the augmentation.
